# Iatrogenic Femoral Arteriovenous Fistula with Pseudoaneurysm Associated with Worsening Heart Failure Years after Percutaneous Impella Placement

**DOI:** 10.1155/2022/7005236

**Published:** 2022-06-24

**Authors:** Andy Y. Wang, Ali Al Jabri, Edward R. Jewell, Angela L. Jellison

**Affiliations:** Vascular Surgery, Lahey Hospital and Medical Center, Burlington, MA, USA

## Abstract

Iatrogenic arteriovenous fistulas (AVFs) and pseudoaneurysms (PSAs) are rare complications that may develop years after vascular access, and high-volume flow through these AVFs have been hypothesized to contribute to chronic heart failure. Formation of an AVF or PSA following Impella placement has rarely been described in the literature. Here, we describe a patient who had percutaneous placement of an Impella ventricular assist device through his right groin three years prior, now presenting with worsening heart failure and symptoms of volume overload. He was discovered to have a new, high-flow common femoral artery to femoral vein AVF with an associated PSA. The AVF and associated PSA were resected and repaired. This case study highlights a rare access-site complication from percutaneous Impella placement associated with worsening heart failure, strategies for preventing this complication during peripheral access, and the need to consider this differential in such a patient with a history of peripheral access who has an unexplained worsening of heart failure.

## 1. Introduction

Blood vessel trauma through peripheral access may result in a rare formation of an arteriovenous fistula (AVF) [1–3]. High-volume flow through such a fistula is thought to potentially exacerbate chronic heart failure [4]. However, this phenomenon has rarely been described in cases of Impella ventricular assist device placement and may be missed in such patients who later develop worsening heart failure. Here, we describe a patient with worsening heart failure found to have a high-flow AVF likely due to access-site injury from prior percutaneous placement of an Impella ventricular assist device.

## 2. Case Presentation

The patient is a 51-year-old male admitted to the hospital with shortness of breath and clinical signs of volume overload from acute exacerbation of chronic heart failure with reduced ejection fraction at around 25-30% as determined via echocardiogram. His past medical history is notable for nonischemic cardiomyopathy (cardiogenic shock and biventricular systolic dysfunction) requiring placement of an Impella ventricular assist device through his right groin three years before presentation, paroxysmal atrial fibrillation on anticoagulation, right lower extremity deep vein thrombosis, chronic kidney disease, an implantable cardioverter defibrillator (ICD), and alcoholic cirrhosis. Careful physical exam revealed a palpable thrill on the right groin distal to the inguinal ligament, which suggested a vascular malformation. This area was the same location where peripheral access was obtained for Impella placement. Information regarding access site management after Impella placement is unavailable.

**Figure 1:**
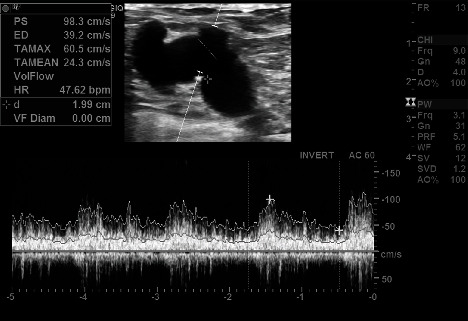
Duplex ultrasound showing an abnormally shaped AV fistula with associated pseudoaneurysm.

Duplex ultrasound was performed for imaging (Figure 1). Of note, we were unable to obtain computed tomography (CT) scan with contrast as the patient had kidney injury and unable to perform Magnetic Resonance Angiography (MRA). Ultrasound imaging estimated the flow through the vein adjacent to the fistula to be approximately 2.8 L/min and revealed an unusual shape suggestive of pseudoaneurysm.

**Figure 2:**
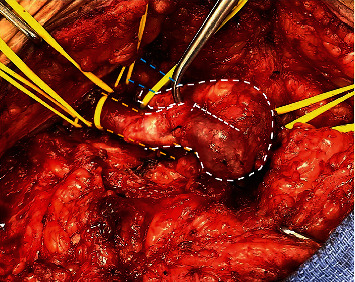
Dissection of the arteriovenous fistula and pseudoaneurysm (white borders). Top left is the inguinal ligament, orange borders highlight the common femoral artery, and blue borders highlight femoral vein.

**Figure 3:**
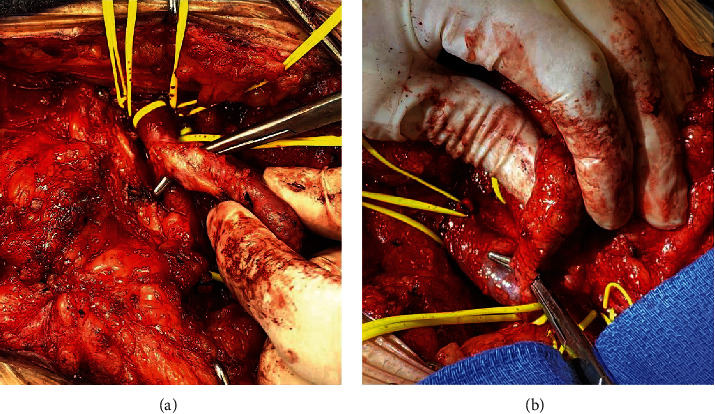
Views of the arterial and venous connections to the structure. (a) Arterial connection to AV fistula and pseudoaneurysm. (b) Venous connection to AV fistula and pseudoaneurysm.

Next, surgery was undertaken to resect the AVF with PSA. Swan-Ganz catheter found pulmonary artery pressures to be elevated. Surgical exposure identified a common femoral artery to femoral vein AVF with PSA, shown in Figure 2, with contributing branches identified (Figures 2 and 3).

**Figure 4:**
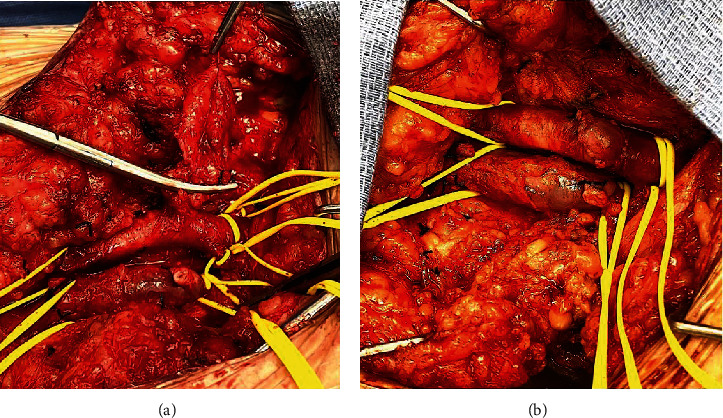
Repair of the AV fistula with pseudoaneurysm. (a) Suture ligation of the venous limb of the structure with AV fistula/pseudoaneurysm remnant. (b) Repaired AV fistula/pseudoaneurysm prior to closing. The common femoral artery is at the top with a great saphenous vein patch sewn in. The femoral vein is at the bottom with the stump ligated.

Proximal control of the femoral artery at the ligament level and control of the vein at the ligament level were established. The AVF and PSA were mobilized along with obtaining distal artery and vein control (Figure 3). The venous end was suture ligated, observing a small opening into the vein (Figure 4). The fistula then divided from the artery and a harvested saphenous vein was sewn to the artery (Figure 4).

## 3. Discussion

We describe an arteriovenous fistula and associated pseudoaneurysm in a patient with worsening heart failure who had percutaneous placement of an Impella ventricular assist device three years prior to presentation of volume overload symptoms. Iatrogenic AVFs and PSAs may be rare complications years after peripheral vascular access, and prior reports have documented delayed formation of AVFs between femoral, subclavian, and radial vessels due to percutaneous vascular access [1–3]. Furthermore, it has been thought that such AV fistulas resulting from such injuries may contribute to worsening heart failure and high pulmonary artery pressures, as seen in this patient [4]. Pseudoaneurysms also have complications such as rupture, distal embolization, local pain, neuropathy, and local ischemia [5]. Risk factors for both AVFs and PSAs involve obesity, location of access site, anticoagulation, hypertension, hemodialysis, and interventional procedures [6, 7].

Formation of an AVF with PSA following Impella placement has rarely been described in the literature. One of the few papers that discuss this briefly mentioned formation of an AVF as a possible access site complication following Impella-supported percutaneous coronary interventions is [8]. However, none of these papers have described such a vascular malformation to lead to worsening of symptoms requiring a hospital admission.

Preventative measures can be taken during peripheral vascular access to avoid development of AVF formation. At the inguinal ligament, the common femoral vein is medial to the common femoral artery. However, the vein travels distally more laterally and posterior as it reaches the edge of the femoral triangle, and a distal access site is more likely to injure either or both the artery and vein to predispose to fistula or pseudoaneurysm formation [7]. To avoid this, the inguinal ligament rather than the femoral crease should be used as a landmark for access to avoid being too distal.^7^ Optimally, the puncture site should be 1-2 cm below the inguinal ligament and above the femoral bifurcation [9]. This could be supplemented with the use of ultrasound guidance for better visualization. Furthermore, vascular closure devices could be used to result in lower complication rates.^9^ Although routine ultrasound follow-up was not performed after Impella placement in our patient, this may be a cost-saving and effective preventative measure to monitor for the complication of AVF formation.

Originally admitted for worsening heart failure symptoms without clear explanation, the patient was only discovered to have an arteriovenous fistula and pseudoaneurysm after closer history detailing Impella placement three years prior and a detailed physical exam revealing a palpable thrill on the right groin in the area of the access site. This highlights an important consideration of the medical history and physical exam in the workup of patients presenting with unexplained symptoms of volume overload who may have an AVF and/or PSA. Medical history should include discussion of prior vascular access and a simple physical exam that searches for any palpable thrills. Positive results can then be followed up with imaging and possible surgical resection as exemplified in this patient.

## 4. Conclusion

In summary, this 51-year-old gentleman presented with volume overload symptoms secondary to heart failure, found to have a femoral AVF and pseudoaneurysm likely due to percutaneous insertion of an Impella ventricular assist device through the right common femoral artery. This is a rare case of an access site complication after Impella placement that has rarely been documented. We describe the link between this high-flow AVF with PSA and exacerbation of heart failure and strategies for preventing this complication as well as the importance of the history and physical exam for diagnosis.

## Data Availability

Patient information is not available due to confidentiality.
